# Quick, eyes! Isolated upper face regions but not artificial features elicit rapid saccades

**DOI:** 10.1167/jov.23.2.5

**Published:** 2023-02-07

**Authors:** Maximilian Davide Broda, Theresa Haddad, Benjamin de Haas

**Affiliations:** 1 Experimental Psychology, Justus Liebig University Giessen, Germany; 2 Center for Mind, Brain and Behavior (CMBB), University of Marburg and Justus Liebig University, Giessen, Germany

**Keywords:** glasses, masks, rapid saccades, eyes, facial features

## Abstract

Human faces elicit faster saccades than objects or animals, resonating with the great importance of faces for our species. The underlying mechanisms are largely unclear. Here, we test two hypotheses based on previous findings. First, ultra-rapid saccades toward faces may not depend on the presence of the whole face, but the upper face region containing the eye region. Second, ultra-rapid saccades toward faces (and possibly face parts) may emerge from our extensive experience with this stimulus and thus extend to glasses and masks – artificial features frequently encountered as part of a face. To test these hypotheses, we asked 43 participants to complete a saccadic choice task, which contrasted images of whole, upper and lower faces, face masks, and glasses with car images. The resulting data confirmed ultra-rapid saccades for isolated upper face regions, but not for artificial facial features.

## Introduction

Faces are a good predictor of human fixation locations in static (End & Gamer, 2017) and dynamic scenes (Rubo & Gamer, 2018; Smith & Mital, 2013). The mere addition of faces to salience models improves predictions based on low-level features, such as orientation, color, or intensity (Cerf, Harel, Einhäuser, & Koch, 2008; Cerf, Paxon Frady, & Koch, 2009). Faces also attract more and longer fixations than other semantic categories (Linka & de Haas, 2020; Xu, Jiang, Wang, Kankanhalli, & Zhao, 2014) or body features (Broda & de Haas, 2022b). When present in a scene, most observers perform a saccade to a face early during exploration (Cerf, Harel, Einhäuser, & Koch, 2008) and with higher velocity compared to inanimate objects (Borovska & de Haas, 2022).

Faces can also elicit saccades with lower latency than other semantic categories (Crouzet, Kirchner, & Thorpe, 2010). This finding is based on the saccadic choice task paradigm, in which participants are simultaneously shown two different stimuli in their left and right visual hemifields. These stimuli stem from different categories, which are defined as target and distractor at the beginning of each block. Participants are asked to initiate a saccade as fast as possible to the target stimulus, as soon as the target and distractor stimuli appear. In trials that contain faces as targets, saccadic latencies can be as low as 100 ms (Crouzet, Kirchner, & Thorpe, 2010). This is significantly faster than observed for other target categories, such as inanimate objects or animals (Boucart et al., 2016; Crouzet & Thorpe, 2011; Guyader, Chauvin, Boucart, & Peyrin, 2017; Kauffmann, Khazaz, Peyrin, & Guyader, 2021; Kauffmann et al., 2019; Little, Jenkins, & Susilo, 2021). Crucially, this processing advantage is robust and exists across the visual field (Boucart et al., 2016), for scrambled (Kauffmann, Khazaz, Peyrin, & Guyader, 2021), orientation- and contrast-inverted (Little, Jenkins, & Susilo, 2021) as well as low-pass filtered faces (Guyader, Chauvin, Boucart, & Peyrin, 2017). Furthermore, faces are highly potent distractors, underlining the strong face bias in humans. Recent research provided evidence that scrambled faces could still elicit ultra-rapid responses (Kauffmann, Khazaz, Peyrin, & Guyader, 2021), showing that holistic face processing is not necessary for rapid saccadic responses. This finding may point to the importance of single face features for early detection. Indeed, recordings from the posterior lateral face patch in macaques show that a single eye in a face outline can elicit fast neural responses (latencies below 50 ms), but this fast response is diminished for faces lacking the contralateral eye (Issa & DiCarlo, 2012). However, it is unclear whether isolated face parts elicit rapid saccades. Although the study by Kauffmann, Khazaz, Peyrin, and Guyader (2021) provides evidence that facial features do not have to be arranged the usual way to elicit rapid saccades, it is unclear whether they can do so when presented in isolation.

A further open question regarding ultra-rapid saccades is how plastic the underlying mechanisms are. Attentional orienting toward faces and face-like stimuli has been found for children of all ages starting from birth (Fantz, 1963; Farroni, et al., 2005; Johnson, Dziurawiec, Ellis, & Morton, 1991; Johnson, Senju, & Tomalski, 2015; Linka, Sensoy, Karimpur, Schwarzer, & de Haas, 2022) and even before (Reid et al., 2017). This may point to innate biases, a possibility further underscored by twin studies finding strong genetic components for the individual tendency to fixate faces in scenes (Constantino, et al., 2017; Kennedy, D'Onofrio, Quinn, Bölte, Lichtenstein, & Falck-Ytter, 2017). However, recent developmental findings suggest that such individual salience biases can be modulated by impactful experience, like learning to read (Linka, Sensoy, Karimpur, Schwarzer, & de Haas, 2022). Similarly, macaques who grew up face deprived tend to fixate faces less, but hands more (Arcaro, Schade, Vincent, Ponce, & Livingstone, 2017). Thus, whereas faces are highly salient stimuli that quickly attract attention (Cerf, Harel, Einhäuser, & Koch, 2008; Crouzet, Kirchner, & Thorpe, 2010) in most observers (Broda & de Haas, 2022b; de Haas, Iakovidis, Schwarzkopf, & Gegenfurtner, 2019), the underlying mechanisms seem malleable at least to some extent. To test whether this extends to rapid saccades, we used artificial features frequently seen as part of a face.

Due to the coronavirus disease 2019 (COVID-19) pandemic, during the past 2 years face masks were mandatory in many public settings in Germany. This entailed massive exposure to faces that were partly covered by masks. Another set of artificial facial features frequently encountered are glasses. It is unclear whether this type of perceptual experience can shape the mechanisms underlying rapid saccades toward faces (and potentially isolated facial features) to also respond to artificial face features. Few previous studies tested whether the face processing system learns to respond to evolutionarily novel features and with conflicting results. One functional magnetic resonance imaging (fMRI) study reported no preferential responses to isolated glasses in the fusiform face area (Axelrod & Yovel, 2011), whereas an electroencephalogram (EEG) study found that glasses (like faces) can evoke N170 responses, suggesting similarities in perceptual processing (Cao, Yang, & Hu, 2016).

Here, we use a saccadic choice task with cars as a control condition to test the potency of isolated facial features as distractors and to elicit rapid saccades, both in comparison to whole faces (Crouzet, Kirchner, & Thorpe, 2010; Kauffmann, Khazaz, Peyrin, & Guyader, 2021). We test both natural and artificial features using depictions of isolated upper and lower face regions, masks, and glasses in different conditions.

## Methods

### Participants

Forty-three healthy adult participants with normal or corrected-to-normal vision took part in the experiment (mean age = 24.77 years, *SD* = 4.32 years; 28 women, and 39 right-handed). The data from three participants were excluded because more than 50% of their trials were invalid (2 participants; see Data analysis) or incorrect (one participant). All participants provided written informed consent and the study was approved by the institutional review board and in accord with the Declaration of Helsinki (except for pre-registration). Participants could choose between course credit or 12 Euros for reimbursement.

### Stimuli

**Figure 1.**
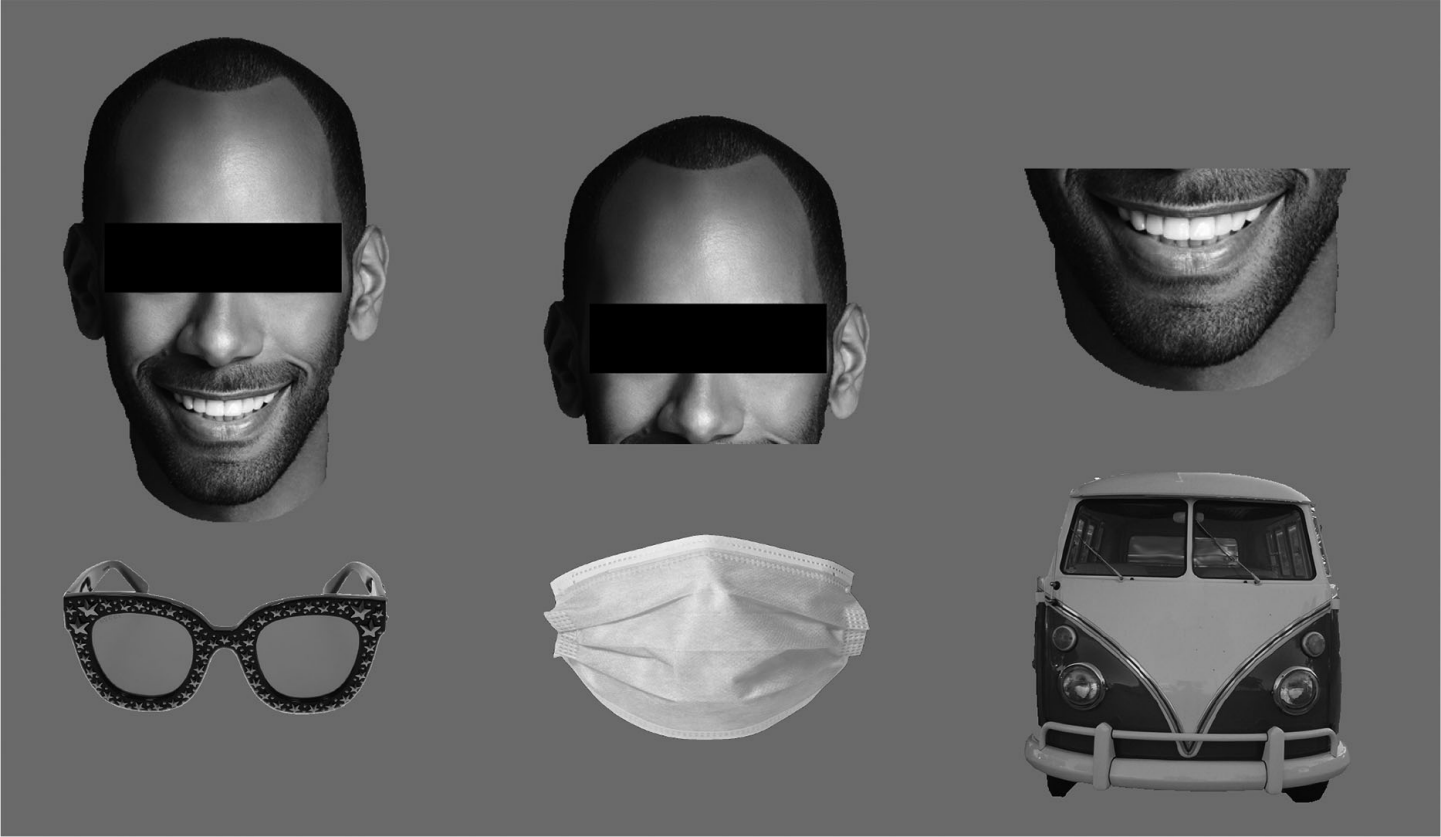
Example stimuli for each condition. Faces, face regions, and cars were adapted from the fLoc functional localizer (Stigliani, Weiner, & Grill-Spector, 2015), glasses and masks were silhouette-cropped manually from various online sources. All stimuli were scaled to a width of 6.9 degrees visual angle. A black bar was added to render the depicted face unrecognizable for display purposes only (participants saw unmodified stimuli in the experiment).

Stimuli consisted of 150 grey scale images of silhouette-cropped cars and 30 silhouette-cropped grey scale images (see Supplementary Table S1 for mean pixel intensities) each from five categories of interest: faces, upper face regions containing the eye region, lower face regions containing the mouth region, glasses, and face masks (Figure 1). Vehicles are an often-used control category (see for example, Crouzet, Kirchner, & Thorpe, 2010; Guyader, Chauvin, Boucart, & Peyrin, 2017; Kauffmann et al., 2019; Kauffmann, Khazaz, Peyrin, & Guyader, 2021) which is why we decided to use images of cars in this experiment. All images of faces and cars were taken from the fLoc functional localizer package (Stigliani, Weiner, & Grill-Spector, 2015; http://vpnl.stanford.edu/fLoc/), images of glasses and masks stem from various online resources and were silhouette-cropped manually. All images were shown on a mid-grey background and scaled to a width of 6.9 degrees visual angle, with their center placed 8 degrees visual angle to the left or right of a central fixation point. Mean image height varied between 2.5 (glasses) and 9.1 (faces) degrees visual angle (Supplementary Table S2).

### Procedure

Participants sat in a dark room with their head in a chin- and forehead-rest approximately 56 cm from the screen. The position of their right eye was tracked at a frequency of 1 kHz with a tower mount Eyelink 1000 (SR Research, Ottawa, Canada). Saccades were detected and recorded online, using default settings of the Eyelink parser (velocity >30 degrees/s, acceleration >9500 degrees/s^2^). Stimuli were shown on a 23.8-inch LG Ultra HD monitor at a resolution of 3840 × 2160 pixels and a refresh rate of 59 Hz. The experiment was programmed using Psychtoolbox version 3.0.16 (Kleiner et al., 2007) in MATLAB R2019a (Mathworks, Natick, MA, USA) on a Windows 10 PC.

The experimental design followed that of Kauffmann, Khazaz, Peyrin, and Guyader (2021). Specifically, in each trial, an image from one category of interest was shown on the left or right of the fixation dot (diameter 0.1 degrees visual angle) and a car image on the other. Participants were either instructed to saccade as fast and accurately as possible to the cars or to the images of a specific category of interest, in separate blocks of 60 trials each. The side of the target was counterbalanced across trials for each block. Each category of interest served as target and distractor once, for a total of 10 blocks per participant (i.e. 600 trials), which were presented in randomized order. The eye tracker was (re-)calibrated before each block using a nine-point grid. Including calibrations and breaks, the experiment lasted 60 to 90 minutes per participant.

In each trial, participants first fixated a central black fixation dot (diameter 0.1 degrees visual angle), which disappeared after a random interval of 0.8 to 1.6 seconds for a gap of 0.2 seconds followed by a target-distractor pair shown for 0.4 seconds and an inter-trial interval of 1 second. Every 10 trials, a larger fixation dot appeared, indicating a self-paced drift check, which gave participants the opportunity to take a short break (without leaving the chin- and head rest). Participants initiated the next set of 10 trials by fixating the larger fixation dot and pressing the space bar. The deviation of the recorded fixation location from the nominal center was recorded. The median deviation across trials was <0.51 degrees visual angle for every participant, confirming excellent calibration accuracy (mean and standard deviation across participants 0.33 and 0.08 degrees, respectively).

### Data analysis

Our aim was to test a potential advantage in terms of accuracy and saccadic reaction times relative to cars for each category to determine whether the advantage observed for faces (e.g. Crouzet, Kirchner, & Thorpe, 2010) generalizes to the other categories. Additionally, we were interested in testing potential differences in performance and saccadic reaction time advantages (versus cars), as well as absolute performance and reaction times between these categories.

To this end, we analyzed the proportion of correct responses and reaction times for first saccades with an amplitude >1 degrees visual angle after stimulus onset. Trials in which this saccade had a latency <50 ms, an initial eccentricity >2 degrees visual angle, or a duration >100 ms were excluded as invalid. The data from two participants with >50% invalid trials and that of an additional participant with >50% incorrect responses were disregarded entirely. There were 81.1% of the remaining trials that were valid and of those 78.88% were correct responses.

For each participant and block (i.e. target-distractor category) we computed the proportion of correct responses as well as the mean saccadic reaction time. Performance and saccadic reaction times which were >3.5 standard deviations above or below the group mean were excluded as outliers (listwise and separately for each ANOVA and *t*-test). Performance advantages and saccadic reaction time differences were calculated as the difference between performance (or reaction time), when a given category of interest served as target versus when it served as distractor. Absolute performance and reaction times as well as advantages were subjected to separate repeated-measures ANOVAs and post hoc *t*-tests in MATLAB (Mathworks, Natick, MA, USA). Significance was determined at a family-wise error rate (FWE) of α = 0.05 using the Holm-Bonferroni method to correct for multiple testing (*p* values are uncorrected, but only reported as significant if they survived FWE correction).

Additionally, we explored minimum saccadic reaction times for each category of interest. For this, we pooled saccadic reaction times across observers and binned them, separately for correct and incorrect responses and each target category. The 30 bins were 10 ms wide and centered 60 to 350 ms post-stimulus onset. For each bin, we used a binomial test to infer whether the proportion of correct responses was significantly higher than expected by chance (*p* < 0.05). The minimum reaction time was defined as the first significant bin followed by at least four more consecutive significant bins (see for example, Kauffmann, Khazaz, Peyrin, & Guyader, 2021). Additionally, we used a permutation test with 10,000 iterations to test whether the proportion of correct responses in the minimum reaction time bin was significantly higher for the fastest condition than the other conditions. Specifically, we first pooled correct and incorrect saccades (with corresponding reaction times) across the two conditions to be compared. In each iteration, we then randomly re-assigned these saccades to either condition and computed the resulting difference in the proportion of correct saccades. This resulted in a null-distribution of differences for the corresponding bin and pair of conditions. The observed difference was deemed significant if it was more extreme than 95% of cases in the null distribution.

## Results

### Performance

**Figure 2.**
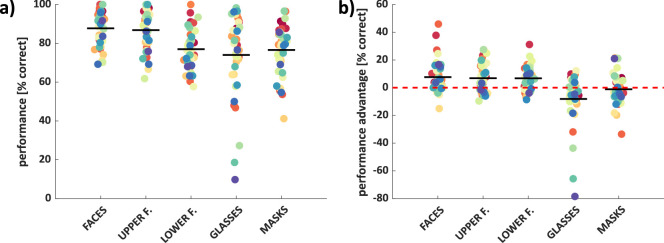
Performance (**a**) and performance advantage (**b**) for each condition. Each dot shows the mean performance (or performance advantage) for one observer, with color tied to observer identity across conditions (repeated measures). *Black horizontal lines* indicate group mean values. Performance corresponds to the proportion of first saccades going to the target in the block in which the respective category served as target. Performance advantage refers to the difference in the proportion of correct saccadic choices in the block in which the respective category served as target versus blocks in which it served as distractor (and cars served as targets).

The mean proportion of correct responses for speeded saccades toward faces, upper face regions, lower face regions, glasses, and masks were 87.8%, 86.8%, 77.0%, 74.0%, and 76.6%, respectively (Figure 2a; Supplementary Figure S1a when they served as distractors). A one-way repeated measures ANOVA revealed a significant main effect of condition *F*(4, 37) = 17.92, *p* < 10^−11^, η^2^ = 0.33, with significantly better performance for the face and upper face regions than all other categories (all *t* > 4.5, *p* < 0.001), and no further significant differences (all *t* < 1.1, *p* > 0.29; Supplementary Figure S2a).

The mean performance advantage for speeded saccades toward faces, upper face regions, lower face regions, glasses, and masks (compared to when they served as distractor) were 7.7% (*t*(38) = 4.10, *p* < 0.001), 6.8% (*t*(39) = 4.17, *p* < 0.001), 6.8% (*t*(39) = 5.09, *p* < 0.001), −8.1% (*t*(38) = −2.73, *p* = 0.010), and −1.1% (*t*(39) = −0.66, *p* = 0.51), respectively (Figure 2b). A one-way repeated measures ANOVA revealed a significant main effect of condition *F*(4, 37) = 12.61, *p* < 10^−8^, η^2^ = 0.25, with a significantly higher advantage for faces, upper face regions, and lower face regions than for glasses and masks (all *t* > 3, *p* < 0.004), and no further significant differences (all *t* < 0.4, *p* > 0.7, but a nonsignificant [ns] trend for masks versus glasses *t*(38) = 1.95, *p* = 0.058; see Supplementary Figure S2b).

### Saccadic reaction times

**Figure 3.**
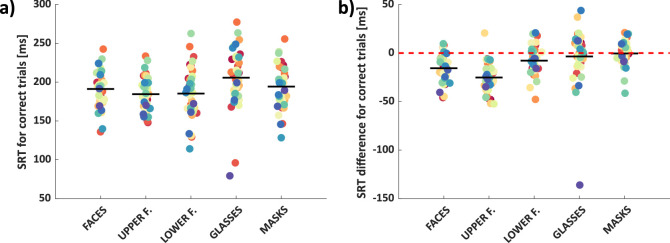
Saccadic reaction time (SRT) (**a**) and SRT advantage (**b**) for each condition. Each dot shows the mean SRT (or SRT advantage) for one observer, with color tied to observer identity across conditions (repeated measures). *Black horizontal lines* indicate group mean values. SRTs correspond to the latency of the first saccade going to the target in correct trials of the block in which the respective category served as target. SRT differences refer to the difference of SRTs of correct trials in the block in which the respective category served as target versus blocks in which it served as distractor (and cars served as targets).

The mean saccadic reaction time of correct responses for speeded saccades toward faces, upper face regions, lower face regions, glasses, and masks were 191 ms, 185 ms, 185 ms, 206 ms, and 194 ms, respectively (Figure 3a; see Supplementary Figure S1b when they served as distractors). A one-way repeated measures ANOVA revealed a significant main effect of condition *F*(4, 38) = 13.62, *p* < 10^−8^, η^2^ = 0.26, with significantly faster reaction times for upper and lower face regions than for masks and glasses (all *t* < −3.3, *p* < 0.002) and significantly faster reaction times for faces compared to glasses (*t*(39) = −3.41, *p* = 0.002; all other *t* < 2.6, *p* > 0.01; see Supplementary Figure S2c).

The mean reaction time difference for speeded saccades toward faces, upper face regions, lower face regions, glasses, and masks (compared to when they served as distractor; negative values meaning faster) were −16 ms (*t*(39) = −7.12, *p* < 0.001), −25 ms (*t*(38) = −10.60, *p* < 0.001), −8 ms (*t*(39) = −3.44, *p* = 0.001), −2 ms (*t*(39) = −0.44, *p* = 0.661), and 0 ms (*t*(39) = −0.22, *p* = 0.829), respectively (see Figure 3b). A one-way repeated measures ANOVA revealed a significant main effect of condition *F*(4, 38) = 13.91, *p* < 10^−8^, η^2^ = 0.27, with a significantly higher reaction time advantage for upper face regions compared to all other categories (including faces and lower face regions; all *t* < −2.8, *p* < 0.008), a higher reaction time advantage for faces compared to lower face regions, glasses, and masks (all *t* < −2.7, *p* < 0.009; all other *t* < 2.4, *p* > 0.02; see Supplementary Figure S2d).

### Minimum saccadic reaction times

**Figure 4.**
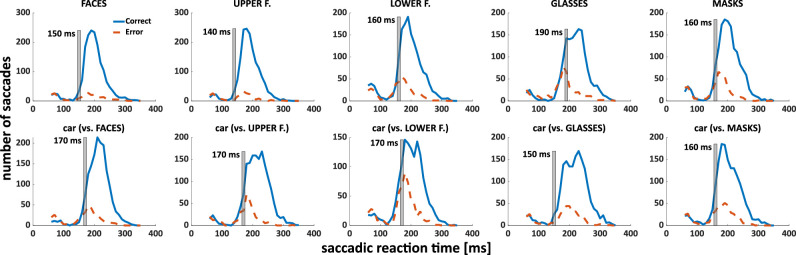
Distribution of saccadic reaction times for correct (*blue*) and incorrect (*dashed red lines*) saccades for each condition. The 10 ms bin from which correct responses significantly exceeded incorrect ones is highlighted with a *grey bar* for each condition. The *top row* shows conditions in which the respective category of interest was target, the *bottom row* the corresponding conditions in which it served as distractor (and cars were targets).

We additionally explored minimum saccadic reaction times, determining the lowest latencies from which the proportion of correct trials became significantly higher than expected by chance (see for example, Material and Method for details). The minimum reaction times for faces, upper face regions, lower face regions, glasses, and masks (versus cars when the respective category served as distractor) were 150 ms (vs. 170 ms), 140 ms (vs. 170 ms), 160 ms (vs. 170 ms), 190 ms (vs. 150 ms,) and 160 ms (vs. 160 ms) respectively (Figure 4). Permutation tests indicated that performance in the 140 ms bin was significantly better for upper face regions compared to lower face regions (*p* < 0.001) and whole faces (*p* = 0.033). This advantage for upper over lower face regions also held in the 150 ms bin (*p* < 0.001).

## Discussion

The current study tested whether ultra-rapid saccades toward faces generalize to isolated natural and artificial facial features. To test this, we used the saccadic choice task, contrasted images of whole, upper and lower faces, face masks, and glasses with car images and compared response times and accuracies for each category.

Our results replicate previous findings, showing significant performance and reaction time advantages for target faces compared to target cars (e.g. Crouzet, Kirchner, & Thorpe, 2010; Kauffmann et al., 2019, Kauffmann, Khazaz, Peyrin, & Guyader,2021). Crucially, they showed similar advantages for isolated upper (and to a lesser degree lower) face regions, but not for glasses or face masks. These differences in performance and reaction time advantages were significant, as became apparent when we directly compared faces and face parts to glasses and masks. Glasses and masks further elicited minimum saccadic reaction times, which were as slow or slower than those for cars, and whole and upper faces. These results suggest that mere exposure and visual expertise (Cao, Yang, & Hu, 2016) do not entail the type of processing advantages seen for natural faces and face parts.

Interestingly, upper faces elicited faster minimum saccadic reaction times and significantly greater saccadic reaction time advantages than lower and even the whole faces they were taken from. This finding resonates with the eye region's prominent importance for face detection (Lewis & Edmonds, 2003; Viola & Jones, 2001) and recognition (Linka, Broda, Alsheimer, de Haas, & Ramon, 2022; Peterson & Eckstein, 2012; Sadr, Jarudi, & Sinha, 2003; Vinette, Gosselin, & Schyns, 2004). When eyes are obscured, observers need significantly more time to detect a face. Covering any other facial feature does not alter face detection (Lewis & Edmonds, 2003). Eyes, but not noses or mouths are sufficient and necessary to elicit preferential and fast neural responses in the posterior lateral face patch of macaques and these neural responses have been speculated to contribute to rapid saccades toward faces in humans (Issa & DiCarlo, 2012). This matches previous data in humans showing that rapid saccades toward faces target the eye region (Kauffmann, Khazaz, Peyrin, & Guyader, 2021) and our finding here, showing that isolated upper face regions elicit faster saccades than lower face regions. This advantage for the upper over the lower face region held for minimum saccadic reaction times and mean reaction time advantages (as compared to cars), but the absolute saccadic reaction times elicited by lower face regions were comparable to those for upper faces. At the same time, there was a specific advantage for the upper over the lower face region for absolute performance, but not for performance advantages as compared to cars. Future studies should test the robustness of the upper face advantage and to which degree it depends on the metric used.

Following previous studies, we centered all stimuli on the horizontal meridian. Recent results suggest that the effect of rapid saccades toward faces holds independent of visual field positioning (Kauffmann et al., 2019; Little, Jenkins, & Susilo, 2021). However, recognition performance for facial features is improved for typical compared to atypical visual field locations (de Haas et al., 2016; de Haas, Sereno, & Samuel Schwarzkopf, 2021; de Haas & Schwarzkopf, 2018). When free viewing faces, eyes usually appear in the upper and mouths in the lower visual field. During speeded saccade tasks towards intact and upright faces, typical landing positions are near the eye region (Kauffmann, Khazaz, Peyrin, & Guyader, 2021). Future research could systematically manipulate vertical stimulus positioning to test whether saccadic reaction times decrease when isolated face parts are presented at typical compared to atypical visual field location. Our present results show that face parts and especially the upper face elicit reaction time and performance advantages comparable to whole faces, even when they are presented at the horizontal meridian.

A further line of research matching the possible importance of the eye region for fast face detection considers the typical contrast pattern formed by a face (Dakin & Watt, 2009). The eye region is generally darker than the rest of the face and thus has high contrast compared to its surrounding features like cheeks, nose, and forehead. Saccade latencies decrease with higher contrast and lower spatial frequencies (Ludwig, Gilchrist, & McSorley, 2004). Low-pass filtered faces still contain contrast information and still elicit rapid saccades (Guyader, Chauvin, Boucart, & Peyrin, 2017). It is tempting to speculate that rapid saccades toward faces rely on the contrast of the eye region and possibly the polarity pattern it forms. However, findings regarding the effects of contrast polarity on rapid face detection have been mixed. Contrast inversion has a detrimental effect on face recognition (Dakin & Watt, 2009) and detection (Lewis & Edmonds, 2003). In line with this, face-selective cells in macaques encode contrast polarity (Ohayon, Freiwald, & Tsao, 2012). However, recent research showed that rapid face-directed saccades are robust to changes in contrast polarity (Little, Jenkins, & Susilo, 2021). Future research is needed to further explore the role of isolated face features in fast face detection and specifically the role of the eye region, its contrast and contrast polarity. Our study provides evidence for the special importance of the upper face region, but this included, for example, the nose and cheeks.

Previous studies used stimuli that were embedded in natural scenes and therefore could use square images (see for example, Crouzet, Kirchner, & Thorpe, 2010; Guyader, Chauvin, Boucart, & Peyrin, 2017; Kauffmann, Khazaz, Peyrin, & Guyader, 2021). Note that this approach was not feasible for our design – there is no natural embedding for isolated face regions and the natural background for masks and glasses typically involves faces. Therefore, we isolated stimuli using silhouette crops, which necessarily resulted in varying aspect ratios. Importantly, the constant stimulus width ensured equal eccentricity for the inner edge and center across all stimuli. Previous studies varied in their control for further low-level attributes of stimuli. They found that the rapid saccade effect of faces holds for both color and gray-scaled images (Kauffmann, Khazaz, Peyrin, & Guyader, 2021) and regardless of whether contrast is equated across images or not (Crouzet, Kirchner, & Thorpe, 2010; Kauffmann, Khazaz, Peyrin, & Guyader, 2021). The images used in the present study were gray-scaled but not matched for luminance or contrast. To test whether mean luminance differences between stimulus categories can explain saccadic reaction times and performance, we quantified average pixel intensities for each category (Supplementary Table S1). Average pixel intensities were generally similar across categories, but highest for masks (which most often are white). However, this contrast to the control condition evidently did not elicit rapid saccades.

Finally, it is unclear to which extent rapid saccades in the saccadic choice paradigm rely on general mechanisms of gaze control. Recently, we found that the individual tendency to fixate faces in scenes is strongly correlated to the tendency to fixate eyes within faces (Broda & de Haas, 2022a; Broda & de Haas, 2022b). Even though most humans tend to fixate close to the eye region within a face (Birmingham, Bischof, & Kingstone, 2008; Foulsham, Cheng, Tracy, Henrich, & Kingstone, 2010; Klin, Jones, Schultz, Volkmar, & Cohen, 2002; Peterson & Eckstein, 2012), significant individual differences exist. Preferred fixation landing positions can be as low as the mouth and prove consistent for complex static scenes (Broda & de Haas, 2022b), portraits (Peterson & Eckstein, 2013), director-cut videos (Broda & de Haas, 2022a), and natural free-viewing situations (Peterson, Lin, Zaun, & Kanwisher, 2016). Future studies should test whether interindividual variability in the saccadic reaction time advantage afforded by faces and eyes is linked to individual differences in face and eye salience. We hypothesize greater reaction time advantages for faces and eyes in the saccadic choice task for observers with a stronger tendency to fixate faces and eyes when free-viewing complex scenes.

In conclusion, we found no evidence for ultra-rapid saccades toward artificial face features which reinforces the special importance of natural faces and their features. Our results further suggest that ultra-rapid saccades toward faces are mostly driven by information from the upper face, most likely the eye region.

## Supplementary Material

Supplement 1

Supplement 2

Supplement 3

Supplement 4
