# Supplementary material for: Quick, eyes! Isolated upper face regions but not artificial features elicit rapid saccades
Source: J Vis. 2023 Feb 7;23(2):5. doi: 10.1167/jov.23.2.5 (PMC9919614; doi:10.1167/jov.23.2.5)
Supplement: Supplement 1 [file jovi-23-2-5_s001.pdf]

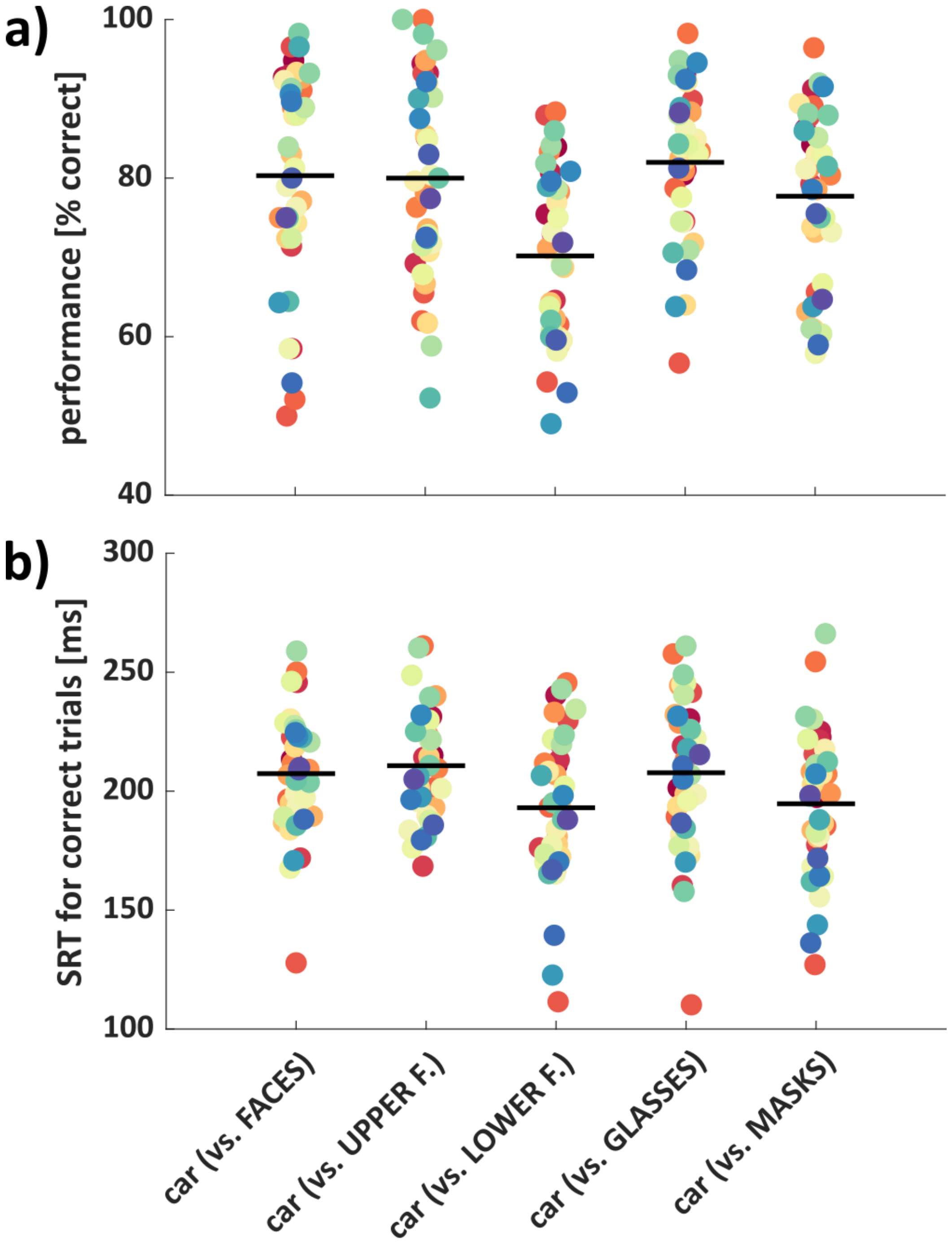

Figure S1. Performance (a) and Saccadic reaction time (SRT, b) for each condition when cars served as target category. Each dot shows the mean performance (or SRT) for one observer, with color tied to observer identity across conditions (repeated measures). Black horizontal lines indicate group mean values. Performance corresponds to the proportion of first saccades going to cars in blocks in which cars served as target. SRTs correspond to saccadic latency towards cars in these correct trials.
