# Supplementary material for: Quick, eyes! Isolated upper face regions but not artificial features elicit rapid saccades
Source: J Vis. 2023 Feb 7;23(2):5. doi: 10.1167/jov.23.2.5 (PMC9919614; doi:10.1167/jov.23.2.5)
Supplement: Supplement 2 [file jovi-23-2-5_s002.pdf]

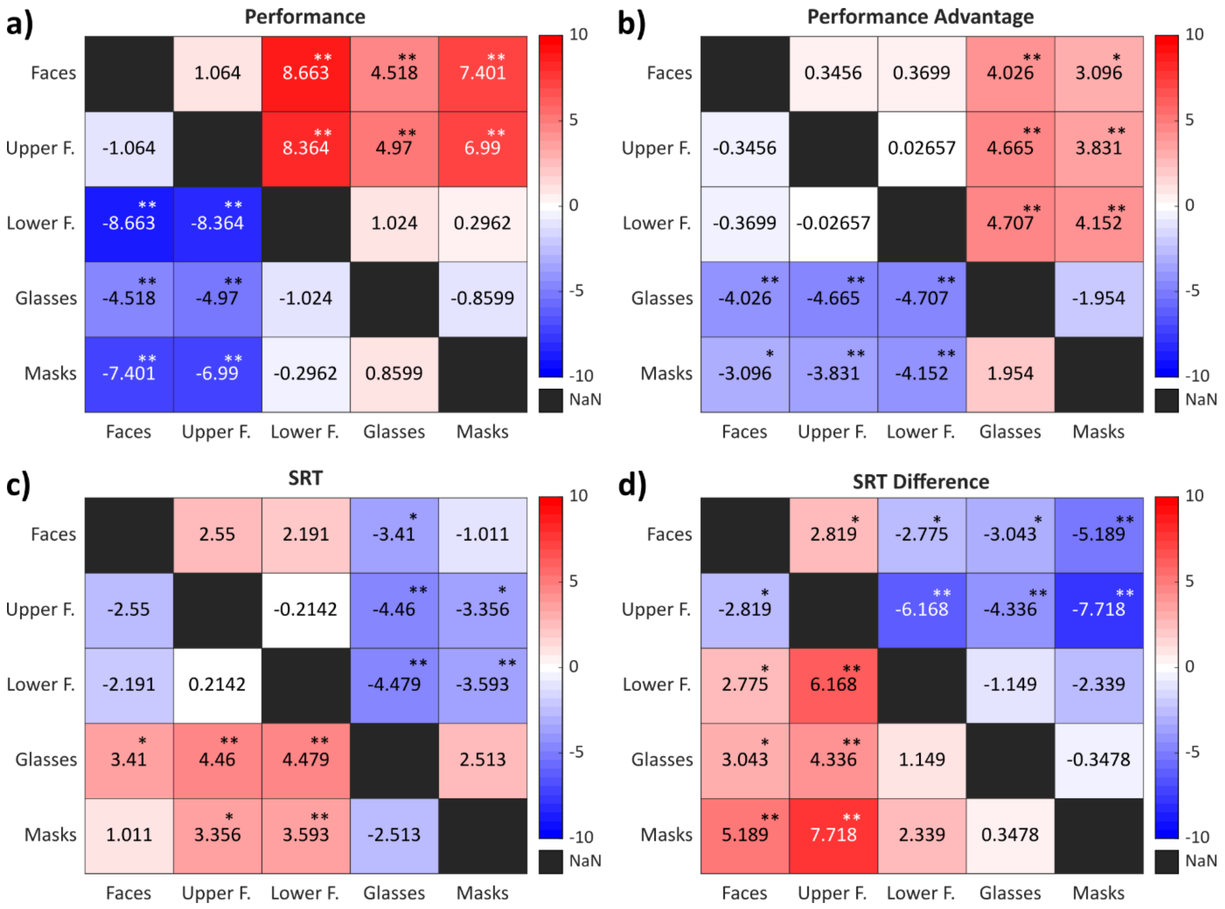

Figure S2. Depiction of all pairwise post-hoc t-tests for (a) performance, (b) performance advantage, (c) SRT and (d) SRT difference. Negative to positive t values are indicated by color and saturation, as shown on the color bar to the right. Each test compares the respective row vs. column feature. That is, positive *t*-values indicate a performance advantage or longer reaction times for the respective row vs. column feature. Asterisks indicate statistical significance (Holm-Bonferroni corrected for 10 tests per dependent variable) ** $p < .001$, * $p < .05$.
