# Supplementary material for: Quick, eyes! Isolated upper face regions but not artificial features elicit rapid saccades
Source: J Vis. 2023 Feb 7;23(2):5. doi: 10.1167/jov.23.2.5 (PMC9919614; doi:10.1167/jov.23.2.5)
Supplement: Supplement 3 [file jovi-23-2-5_s003.pdf]

Table S1 Mean (SD) category pixel intensity.

Faces	***Upper F.***	***Lower F.***	***Glasses***	***Masks***	***Cars***
110.5 (27.4)	108.8 (27.9)	117.4 (33.2)	108.9 (40.8)	145.1 (65.8)	105.7 (33.1)
