# Supplementary material for: Quick, eyes! Isolated upper face regions but not artificial features elicit rapid saccades
Source: J Vis. 2023 Feb 7;23(2):5. doi: 10.1167/jov.23.2.5 (PMC9919614; doi:10.1167/jov.23.2.5)
Supplement: Supplement 4 [file jovi-23-2-5_s004.pdf]

Table S2 Mean (SD) vertical feature size in degrees visual angle.

Faces	***Upper F.***	***Lower F.***	***Glasses***	***Masks***	***Cars***
9.1 (1.3)	6.2 (0.9)	3.5 (0.9)	2.5 (0.3)	4.5 (0.0)	3.4 (1.0)
